# Population dynamics, pathogen detection and insecticide resistance of mosquito and sand fly in refugee camps, Greece

**DOI:** 10.1186/s40249-020-0635-4

**Published:** 2020-03-18

**Authors:** Emmanouil Alexandros Fotakis, Ioannis Apostolou Giantsis, Javier Castells Sierra, Filianna Tanti, Sofia Balaska, Konstantinos Mavridis, Sofoklis Kourtidis, John Vontas, Alexandra Chaskopoulou

**Affiliations:** 1 Department of Crop Science, Pesticide Science Lab, Agricultural University of Athens, Athens, Greece; 2 Institute of Molecular Biology and Biotechnology, Foundation for Research and Technology Hellas, Heraklion, Crete Greece; 3 European Biological Control Laboratory, USDA-ARS, Thessaloniki, Greece; 4 Department of Biology, National and Kapodistrian University of Athens, Athens, Greece; 5 General Directorate of Public Health and Social Welfare, Region of Central Macedonia, Thessaloniki, Greece

**Keywords:** Refugee camp, Mosquito, Sand fly, Insecticide resistance

## Abstract

**Background:**

As of 2015 thousands of refugees are being hosted in temporary refugee camps in Greece. Displaced populations, travelling and living under poor conditions with limited access to healthcare are at a high risk of exposure to vector borne disease (VBD). This study sought to evaluate the risk for VBD transmission within refugee camps in Greece by analyzing the mosquito and sand fly populations present, in light of designing effective and efficient context specific vector and disease control programs.

**Methods:**

A vector/pathogen surveillance network targeting mosquitoes and sand flies was deployed in four temporary refugee camps in Greece. Sample collections were conducted bi-weekly during June–September 2017 with the use of Centers for Disease Control (CDC) light traps and oviposition traps. Using conventional and molecular diagnostic tools we investigated the mosquito/sand fly species composition, population dynamics, pathogen infection rates, and insecticide resistance status in the major vector species.

**Results:**

Important disease vectors including *Anopheles sacharovi*, *Culex pipiens*, *Aedes albopictus* and the *Leishmania* vectors *Phlebotomus neglectus*, *P. perfiliewi* and *P. tobbi* were recorded in the study refugee camps. No mosquito pathogens (*Plasmodium* parasites, flaviviruses) were detected in the analysed samples yet high sand fly *Leishmania* infection rates are reported*. Culex pipiens* mosquitoes displayed relatively high knock down resistance *(kdr*) mutation allelic frequencies (ranging from 41.0 to 63.3%) while *kdr* mutations were also detected in *Ae. albopictus* populations, but not in *Anopheles* and sand fly specimens. No diflubenzuron (DFB) mutations were detected in any of the mosquito species analysed.

**Conclusions:**

Important disease vectors and pathogens in vectors (*Leishmania* spp.) were recorded in the refugee camps indicating a situational risk factor for disease transmission. The *Cx. pipiens* and *Ae. albopictus kdr* mutation frequencies recorded pose a potential threat against the effectiveness of pyrethroid insecticides in these settings. In contrast, pyrethroids appear suitable for the control of *Anopheles* mosquitoes and sand flies and DFB for *Cx. pipiens* and *Ae. albopictus* larvicide applications. Targeted actions ensuring adequate living conditions and the establishment of integrated vector-borne disease surveillance programs in refugee settlements are essential for protecting refugee populations against VBDs.

## Background

From 2015 onwards a major refugee crisis has been developing on Europe’s doorstep. Political instability, totalitarian state-governance, poverty and armed conflict have led to mass displacement of populations (refugees/asylum seekers and immigrants) mainly from African and Asian countries seeking refuge in Europe. Greece is amongst the European countries most widely affected by the ongoing crisis. In 2015 and 2016 over 1 000 000 refugees/immigrants arrived in Greece. Currently over 135 000 refugees are hosted in the country [1, 2], the majority in temporary accommodation sites/refugee camps with varied levels of infrastructures, housing conditions and amenities. Displaced populations travelling and living under poor conditions with limited access to healthcare are at a high risk of exposure to vector borne disease (VBD) infections [3–5]. The risk for VBD transmission is augmented in a context of host country disease endemicity (or high prevalence of disease vectors), inadequate or absent vector control in host regions, and VBD endemicity in the refugee travel route regions and countries of origin.

Greece displays an intense history of VBDs, posing a situational risk factor for disease transmission among vulnerable refugee populations. West Nile virus (WNV) lineage two outbreaks, attributed to *Culex pipiens* mosquitoes [6, 7] have taken place from 2010 onwards with the largest occurring in 2018, resulting in 311 cases and 47 deaths [8]. Autochthonous malaria transmission, though eradicated in the 1970s re-appeared in 2009 reaching a peak of 42 locally acquired cases in 2011 [9]. From 2009 onwards imported and locally acquired *Plasmodium vivax* and *Plasmodium falciparum* cases are reported every year. An added public health threat and a considerable nuisance problem is the wide spread distribution of the invasive mosquito *Aedes albopictus*, a known vector of Chinkungunya and dengue virus [10]. Last but not least, visceral leishmaniasis, a lethal disease if left untreated, vectored by Phlebotomine sand flies is endemic in nearly all geographical areas of the country with *Leishmania infantum* implicated as the predominant etiological agent [11]. The disease is most prevalent in dogs with a few sporadic human cases every year [12].

In the absence of protective human vaccines against most VBDs (including malaria, WNV and leishmaniasis), protection from the diseases largely relies on vector control programs utilizing chemical insecticides to manage vector populations [13]. The larvicides *Bacilus thuringiensis israelensis* (B.t.i.), diflubenzuron (DFB) and pyrethroid based adulticides constitute the main mosquito control insecticide regime in Greece under the current regulatory policies, with the latter insecticides (pyrethroids) also applied in a number of refugee camps.

A worldwide problem associated with the extensive use of a limited number of available insecticides in vector and agricultural pest control is the development of insecticide resistance, threatening the effectiveness of control efforts. Resistance is primarily conferred by point mutations on the insecticide’s target molecule (target site resistance) and/or increased activity of detoxification enzymes (metabolic resistance) [14]. Insecticide resistance has been reported in important vector species in Greece and neighbouring countries. Voltage gated sodium channel (*VGSC*) mutations (*kdr* mutations) associated with pyrethroid resistance have been detected at high frequencies (reaching 88.3%) in *Cx. pipiens* mosquitoes from Northern and Central Greece [15]. Presence of the *kdr* mutation L1014F was recently also monitored in sand fly populations from Turkey and Greece (including specimens sampled from Souda refugee camp in Chios island) and was detected in the major *Leishmania* vector *Phlebotomus papatasi* in Turkey [16]. Striking DFB resistance, attributed to the chitin synthase (*CHS*) mutations I1043L and I1043F, was reported in *Cx. pipiens* populations from Northern Italy in 2016–2017 [17, 18]. While the *VGSC* mutations V1016G, I1532T, F1534C/L associated with pyrethroid resistance in *Ae. albopictus* have been recorded in *Ae. albopictus* populations from Italy and Greece where the mutation 1534C was detected at a frequency of 24.2% in the Athens population [19, 20].

Integrated vector/pathogen surveillance is of utmost importance in epidemiological settings of increased public health significance such as refugee camps. Refugee settlements, whether temporary or permanent, represent unique ecological settings which inevitably affect and are affected by the surrounding ecosystem. Within these unique, vulnerable communities, the status of arthropod vectors remains unknown. Fine scale entomological information on vector species composition and seasonal abundance, vector insecticide resistance status and the presence of arthropod-borne pathogens is essential information in order to evaluate the risk for VBD transmission within these communities and design effective and efficient context specific vector and disease control programs (evidence based control). A vector/pathogen surveillance network targeting mosquitoes (Diptera: Culicidae) and sand flies (Diptera: Psychodidae: Phlebotominae) was deployed in temporary refugee camps in Greece in 2017. Preliminary findings of the study relating to sand fly species composition and *Leishmania* circulation were communicated in reference [21]. The present study compiles the full data produced by the surveillance network relating to sand fly and mosquito species composition, population dynamics, pathogen infection rates, and insecticide resistance status in four major refugee camps in Greece.

## Materials and methods

### Study sites

**Fig. 1:**
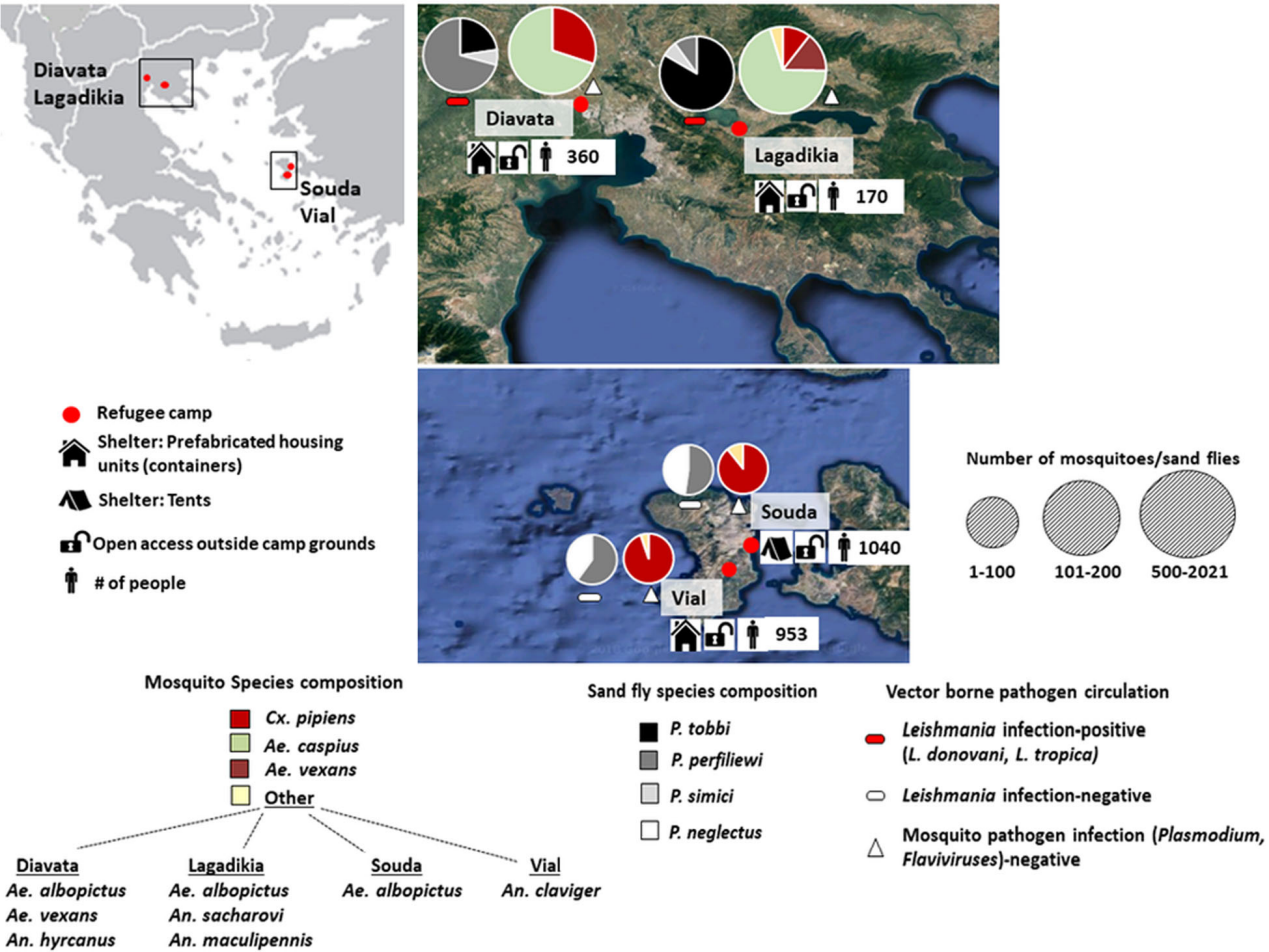
Vector composition and vector borne pathogen detection in Refugee camps, Greece In each refugee camp site - 2 chart pies are depicted. From left to right (in each site); Sand fly species composition chart pie, followed by Mosquito species composition chart pie. Sand fly species data displayed corresponds to male and female^(^*^)^ adult collections, mosquito species data corresponds to adult female collections (under representation of *Aedes albopictus* specimens/egg collections not included). Mosquito and sand fly species recorded are depicted with different colours. The size of each chart pie correlates to the number of sand flies (♂/♀) and mosquitoes (♀) collected in each site. Absence/presence of mosquito and sand fly pathogens^(^**^)^ is depicted with different symbols. Coloured symbols denote infection presence/detection, white colour denotes absence, in the analysed samples. Camp descriptors including shelter type, access status and number of occupants are displayed for the year 2017 [22]. ^(^*^)^The female sand fly specimens identification and ^(^**^)^*Leishmania* detection was conducted in [21]

Four refugee camps were surveyed in 2017: Lagadikia and Diavata camps (open reception facilities), situated in Thessaloniki regional Unit, Central Macedonia, Northern Greece; Vial camp (reception and identification centre) and Souda camp (open reception facility), located on the island of Chios, Northeastern Aegean island complex. Lagadikia camp is situated 46 km east of Thessaloniki city in an agricultural zone and at 5 km from Lake Koroneia. Diavata camp is situated in a peri-urban setting 7 km away from the agricultural land (20 000 ha of rice-fields) of West Thessaloniki and 2 km away from Gallikos river bed. Vial camp is located in an inland agricultural area of the island with no major water bodies, and Souda camp (currently dismantled) was established in the moat of Chios Castle in Chios town, next to the island’s main harbour. The average size of the four study camps was 1.75 ha. In 2017 the four camps hosted populations mainly from Syria, Afghanistan and Iraq [22]. In the summer of 2017 stray dogs were consistently present in the Thessaloniki camps (Diavata, Lagadikia). Shelter and occupancy information for 2017 [22] is included in Fig. 1.

### Vector surveillance and sample handling

Entomological surveillance was conducted bi-weekly from June to September 2017 with the use of Centers for Disease Control (CDC) light traps baited with dry ice (targeting adult mosquitoes and sand flies) and oviposition traps (for surveillance of the container breeder *Ae. albopictus*). Overall seven sampling events were conducted in each refugee camp with the deployment of three CDC light traps (from 18.00 pm–8.00 am) and four oviposition traps (renewed every 14 days) per site per sampling event. In Lagadikia and Diavata camps (Thessaloniki), all sampling events were conducted within the camp grounds whereas in Souda and Vial camps (Chios) the majority of sampling events (five out of seven in each camp) were conducted in a buffer zone of < 200 m around the camp perimeter.

Mosquitoes collected with CDC light traps were either killed with a twenty minute exposure period at − 20 °C, morphologically identified to species level [13, 23] and stored in 70% ethanol (for use in molecular diagnostic assays, e.g. species identification, resistance mutations and *Plasmodium* parasite detection), or anesthetized with a cold shock and following morphological identification stored in RNA later (for *Flavivirus* detection). Mosquito eggs collected with the oviposition traps were stored at − 20 °C. Sand fly handling, described in reference [21] included dissection of the head from the body in the female wild caught specimens and storage of all body parts in ethanol.

### DNA and RNA extraction

Genomic DNA was extracted from single female adult mosquitoes and from pools of mosquito eggs (five eggs per pool) using the DNAzol protocol according to the manufacturer’s instructions (Invitrogen, Carlsbad, USA). Total RNA was isolated from *Cx. pipiens* mosquito pools (5–10 mosquitoes per pool) using the TRI reagent protocol (Invitrogen, Carlsbad, USA). The purity and concentration of the total extracted RNA from each pool was determined using a NanoDrop 2000c spectrophotometer (Thermo Scientific, Waltham, Massachusetts) and its integrity was confirmed with 1% agarose gel electrophoresis. DNA extraction from individual female sand fly specimens and sand fly species molecular identification was conducted and reported in reference [21]. DNA was extracted using the NucleoSpin Tissue kit (Macherey-Nagel, Düren, Germany) according to the manufacturer’s instructions. Female sand flies were identified to species using a multiplex diagnostic assay based on species-specific single-nucleotide polymorphisms of the nuclear 18S rRNA gene [24]. The male sand flies were identified morphologically to species level.

### Mosquito species molecular identification

A subset of mosquitoes belonging to the *Cx. pipiens* complex were identified to species with a PCR diagnostic assay which relies on polymorphisms in the second intron of the acetylcholinesterase-2 (*AChE2*) locus as described in reference [25]. Primers specific for *Cx. pipiens*, *Cx. quinquefasciatus*, *Cx. pallens*, *Cx. australicus*, and *Cx. torrentium* were included in the assay (Table 1). DNA fragments were analyzed by electrophoresis on a 1.5% agarose gel.

**Table 1:** Molecular assays used in the study: primers and PCR conditions

	Species	Assay	Primers (5′ → 3′)	PCR condition	Reference
Insecticide resistance assays	*Culex pipiens*	*VGSC* (1014F)/*kdr* - allele specific PCR	Cgd1: GTGGAACTTCACCGACTTC Cgd2: GCAAGGCTAAGAAAAGGTTAAG Cgd3: CCACCGTAGTGATAGGAAATTTA Cgd4: CCACCGTAGTGATAGGAAATTTT	95 °C 5 min, 40 cycles × (94 °C 30 s, 48 °C 30 s, 72 °C 1 min), 72 °C 10 min	[26]
	*Cx. pipiens*	*CHS* (1043 L) - allele specific PCR	External_F: GCAGTCCTTCGGCGATCTT External_R: GAACAGTCCGGCGATGGATA ATC_ R: AACAGCAAGTACATAGACGGGAT CTC_ F: GGCTTGATCTACCTGCTGTCTC	95 °C 5 min, 28 cycles × (95 °C 30 s, 68 °C 30 s, 72 °C 1 min), 72 °C 5 min	[17]
	*Cx. pipiens*	*CHS* (1043 M) - PCR and RFLP	Diagnostic I1043M_R: CCCAGGAGACGACGTTCAG Diagnostic I1043M_F: GCCTGTCTCCATCCGCAAG	95 °C 5 min, 30 cycles × (95 °C 30 s, 60 °C 30 s, 72 °C 30 s),72 °C 5 min	[17]
	*Aedes albopictus*	*VGSC* (1016G)/*kdr* - PCR and sequencing	F1016: TTCACCGACTTCATGCACTC R1016: CGCAATCTGGCTTGTTAACTT	95° 3 min, 40 cycles × (94 °C 30 s, 55 °C 30 s, 72 °C 1 min), 72 °C 5 min	–
	*Ae. albopictus*	*VGSC* (1532 T,1534C)/*kdr* - PCR and sequencing	For: GAGAACTCGCCGATGAACTT Rev.: GACGACGAAATCGAACAGGT Rev. for sequencing: AGCTTTCAGCGGCTTCTTC	95 °C 3 min, 40 cycles × (94 °C 30 s, 57 °C 30 s, 72 °C 1 min), 72 °C 5 min	–
	*Ae. albopictus*	*CHS* (1043 L/M) - PCR and sequencing	kkv F3: TCGGAAGTCCTTCGGCTTAT TC kkv R3: TGGATACTTCAATGGAACCTTCC	95 °C 5 min, 40 cycles × (94 °C 30 s, 55 °C 30 s, 72 °C 1 min), 72 °C 10 min	–
	*Anopheles* (*An. sacharovi, An. hyrcanus, An. maculipennis, An. claviger*)	VGSC (1014F/C/S) / *kdr* - PCR and sequencing	KDRF: GGMGAATGGATYGAATCMATGTGGGA KDR R2: GATGAACCRAAATTKGACAAAAGCAA	94 °C 5 min, 35 cycles × (94 °C 1 min, 50 °C 2 min, 72 °C 2 min), 72 °C 2 min	–
	Sand flies (*Phlebotomus perfiliewi, P. tobbi, P. simici, P. neglectus*)	VGSC (1011, 1014, 1016, and 1020) / PCR and sequencing	Vssc8F: AATGTGGGATTGCATGCTGG Vssc1bR: CGTATCATTGTCTGCAGTTGGT	95 °C 5 min, 36 cycles × (94 °C 45 s, 51 °C 50 s, 72 °C 50 s),72 °C 7 min	[27]
Species id assays	*Cx. pipiens* complex	*AChE2*- PCR diagnostic assay	ACEquin: CCTTCTTGAATGGCTGTGGCA ACEpall: ATGGTGGAGACGCATGACG ACEpip: GGAAACAACGACGTATGTACT ACEtorr: TGCCTGTGCTACCAGTGATGTT B1246s: TGGAGCCTCCTCTTCACGG	94 °C 5 min, 35 cycles × (94 °C 30 s, 55 °C 30 s, 72 °C 1 min), 72 °C 5 min	[25]
	*Cx. pipiens* biotype	*CQ11* - allele specific PCR	pipRev: CATGTTGAGCTTCGGTGAA molRev: CCCTCCAGTAAGGTATCAAC biotype-forward: GATCCTAGCAAGCGAGAAC	95 °C 5 min, 40 cycles × (94 °C 30 s, 54 °C 30 s, 72 °C 40 s), 72 °C 5 min	[28]
	*Anopheles*	*ITS2* - PCR and sequencing	5.8 s primer: TGTGAACTGCAGGACACATG 28 s primer: ATGCTTAAATTTAGGGGGTA	94 °C 2 min, 40 cycles × (94 °C 30 s, 53 °C 30 s, 72 °C 50 s), 72 °C 10 min	[29]
	*Anopheles*	*COI* - PCR and sequencing	Cl -N-2191: CCCGGTAAAATTAAAATATAAACTTC Cl-J-1718: GCAGGATTTGGAAATTGATTAGTTCC	94 °C 2 min, 40 cycles × (94 °C 30 s, 50 °C 30 s, 72 °C 50 s), 72 °C 10 min	[29]
	*Ae. albopictus*	*ITS2* - PCR and sequencing	5.8 s primer: TGTGAACTGCAGGACACATG 28 s primer: ATGCTTAAATTTAGGGGGTA	94 °C 10 min, 40 cycles × (94 °C 1 min, 50 °C 1 min, 72 °C 1 min), 72 °C 10 min	[30]
	Sand flies	Direct multiplex PCR	Forward: TGCGGTTAAAACGTTCGTAG perf: TAAACCCACAAATCAGAAT sim: CTTTCATTAAATTTAGCCTGCC papat: TAACATAAGGGGCGTATTAATATGCTTT tob: GCGTRTTAACAATAGAGTCCATTAAA	95 °C 5 min, 32 cycles × (94 °C 40 s, 54 °C 30 s, 72 °C 1 min), 72 °C 10 min	[24]
Pathogen detection assays	*Plasmodium* (*P. vivax, P. falciparum*)	PCR 1 (Nested PCR)	rPLU5: CCTGTTGTTGCCTTAAACTTC rPLU6: TTAAAATTGTTGCAGTTAAAACG	95 °C 5 min, 24 cycles × (94 °C 1 min, 57 °C 2 min,72 °C 2 min), 72 °C 5 min	[31]
		PCR 2 (Nested PCR)	VIV-1: CGCTTCTAGCTTAATCCACATAACTGATAC VIV-2: ACTTCCAAGCCGAAGCAAAGAAAGTCCTTA FAL-1: TTAAACTGGTTTGGAAAACCAAATATATT FAL-2: ACACAATGAACTCAATCATGACTACCCGTC	95 °C 5 min, 30 cycles × (94 °C 1 min, 57 °C 2 min, 72 °C 2 min), 72 °C 5 min	[31]
	*Flavivirus* detection	Conventional one-step reverse transcriptase-PCR (RT-PCR)	Forward: TACAACATGATGGGAAAGAGAGAGAA Reverse: GTGTCCCAGCCGGCGGTGTCATCAGC	50 °C 30 min, 95 °C 3 min, 40 cycles × (95 °C 15 s, 55 °C 20 s, 72 °C 60 s), 72 °C 10 min	[32]
	West Nile virus detection	Multiplex Real Time one step RT-PCR *Taq*Man assay	Forward: GTGATCCATGTAAGCCCTCAGAA Reverse: GTCTGACATTGGGCTTTGAAGTTA FAM probe (MGB): AGGACCCCACATGTT HEX probe (MGB): AGGACCCCACGTGCT	50 °C 15 min, 95 °C 3 min, 45 cycles × (95 °C 3 s and 60 °C 30 s)	[33, 34]

*VGSC* Voltage gated sodium channel; *CHS* Chitin synthase; *kdr* Knock down resistance; *AChE2* Acetylcholinesterase-2; *CQ11* Microsatellite locus; *ITS2* Internal transcribed spacer two; *COI* Cytochrome oxidase subunit 1; *[REF]* References

*Cx. pipiens* mosquitoes molecularly identified to species were also identified to biotype (*pipiens*, *molestus*, hybrid) with the use of a PCR diagnostic assay [28] which relies on polymorphisms of the 5′ flanking region of the microsatellite locus *CQ11* specific for pipiens and molestus alleles. Primers are described in Table 1. DNA fragments were analyzed by electrophoresis on a 2% agarose gel.

In order to verify the species identification in samples morphologically identified as *Ae. albopictus* and in eggs collected from the oviposition traps, the nuclear ribosomal spacer gene, internal transcribed spacer two (*ITS2*) was amplified [30]. Adult specimens were analysed individually while eggs were pooled prior to DNA extraction. DNA fragments were analyzed by electrophoresis on a 1.5% agarose gel, (primers described in Table 1).

All *Anopheles* mosquitoes collected were identified to species with *ITS2* and cytochrome oxidase subunit 1 (*COI*) product sequencing and BLAST analysis.

DNA fragments were analyzed by electrophoresis on a 1.5% agarose gel (primers in Table 1).

Prior to sequencing the PCR products were purified using a commercially available kit (Macherey Nagel, Dueren, Germany). Species identification of mosquitoes belonging to the *Anopheles maculipennis* complex was further confirmed with restriction fragment length polymorphism (RFLP) assays [29] based on *ITS2* and *COI* product restriction sites for the restriction enzymes *Alu*I and *Hpa*II, respectively.

### Pathogen detection

Monitoring of *P. vivax* and *P. falciparum* infections in *Anopheles* mosquitoes was conducted with a genus- and species-specific nested polymerase chain reaction [31]. Genomic DNA extracted from *Anopheles* specimens infected with *P. vivax* and *P. falciparum* were used as positive controls (primers in Table 1).

Conventional one-step reverse transcriptase-PCR (RT-PCR) was performed to assess the presence of flaviviruses based on the method described in reference [32] and detection of WNV was performed by a multiplex real time one step RT-PCR *Taq*Man assay that simultaneously detects and differentiates WNV-lineage 1 and WNV-lineage 2, as described in references [33, 34] (primers in Table 1). All the *Cx. pipiens* specimens which were transported live to the laboratory (*n* = 40, from Diavata and Lagadikia camp) were pooled per camp site and included in the *Flavivirus*/WNV detection analysis. *Leishmania* detection and *Leishmania* species identification were conducted in reference [21]. *Leishmania* presence was evaluated by amplification of a 300–350-bp fragment of the *Leishmania* DNA ribosomal internal transcribed spacer one (ITS1) [35]. *Leishmania* species was identified with a series of assays including; an *ITS1* RFLP assay [36], *ITS1* product sequencing and BLAST analysis, a species-specific PCR assay for the cysteine protease b gene [37] and amplification/sequencing of a segment of the heat-shock protein 70 [38].

### Insecticide resistance genotyping

Presence and frequency of *kdr* mutation L1014F (TTA to TTT) on the *VGSC* was monitored in *Cx. pipiens* mosquitoes with an allele specific PCR assay as described in reference [26] (primers in Table 1). DNA fragments of the *VGSC* gene flanking the codon 1014 in *Anopheles* specimens (*An. sacharovi, An. maculipennis, An. hyrcanus, An. claviger*) and the codons 1016, 1532, 1534 in *Ae. albopictus* samples were amplified, purified and sequenced (primers in Table 1). In sand flies, a DNA fragment of the *VGSC* gene (flanking the codons 1011, 1014, 1016, and 1020) was amplified, purified (Macherey Nagel, Dueren, Germany commercial kit) and sequenced (primers in Table 1) as described in reference [27].

The presence of the chitin synthase C-terminus target site resistance mutations I1043L and I1043M were monitored in *Cx. pipiens* with an allele specific PCR assay and a PCR-RFLP diagnostic assay, respectively, as described in reference [17] (primers in Table 1). A sequence of *Ae. albopictus CHS* gene flanking the codon 1043 was amplified and sequenced for the detection of DFB resistance mutations.

## Results

### Vector species population structure and dynamics

Mosquito and sand fly surveillance conducted in the refugee camps resulted in the collection of important proven and suspected disease vectors (Fig. 1, Table 2).

**Table 2:** Mosquito and sand fly population structure in the study refugee camps

	Mosquitoes										Sand flies				
Refugee camp	*Culex*	*Aedes*				*Anopheles*					*Phlebotomus*				
	*Cx. pipiens*	*Ae. albopictus*		*Ae. caspius*	*Ae. vexans*	*An. sacharovi*	*An. maculipennis*	*An. hyrcanus*	*An. claviger*	Total	*P. tobbi*	*P. simici*	*P. perfiliewi*	*P. neglectus*	Total
		(A)	(E)												
Diavata	606 (31)	-	64 (15)	1409	2	-	-	4 (4)	-	2085	36 (15)	11 (7)	114 (45)	-	161
Lagadikia	44 (25)	8 (8)	75 (15)	402	62	4 (4)	6 (6)	-	-	601	128 (45)	10 (6)	15 (9)	-	153
Souda	84 (18)	9 (9)	23 (15)	-	-	-	-	-	-	116	-	-	13 (11)	11 (10)	24
Vial	18 (12)	-	-	-	-	-	-	-	1 (1)	19	-	-	33 (24)	24 (16)	57

The primary WNV vector *Cx. pipiens* was amongst the most dominant mosquito species in all four camps. Biotype analysis in a sub-sample of the *Cx. pipiens* samples detected the *Cx. pipiens pipiens* biotype as the most dominant in Diavata camp while *Cx. pipiens molestus* was the most dominant in Lagadikia, Souda and Vial camp. The hybrid *Cx. pipiens pipiens/molestus* was recorded in all four camps (Table 3).

**Table 3:** *Culex pipiens* biotype composition in the study refugee camps

			*Culex pipiens* biotype *n*(%)		
Region	Camp	*N*	*pipiens*	*molestus*	hybrid
Thessaloniki	Diavata	29	19 (65.5)	2 (6.9)	8 (27.6)
	Lagadikia	24	2 (8.3)	19 (79.2)	3 (12.5)
Chios	Souda	15	0 (0.0)	11 (73.3)	4 (26.7)
	Vial	9	1 (11.1)	5 (55.6)	3 (33.3)

*N* is the number of samples tested per camp, *n* = the number of samples identified for each biotype, [%] = the corresponding frequency of each biotype per camp

**Fig. 2:**
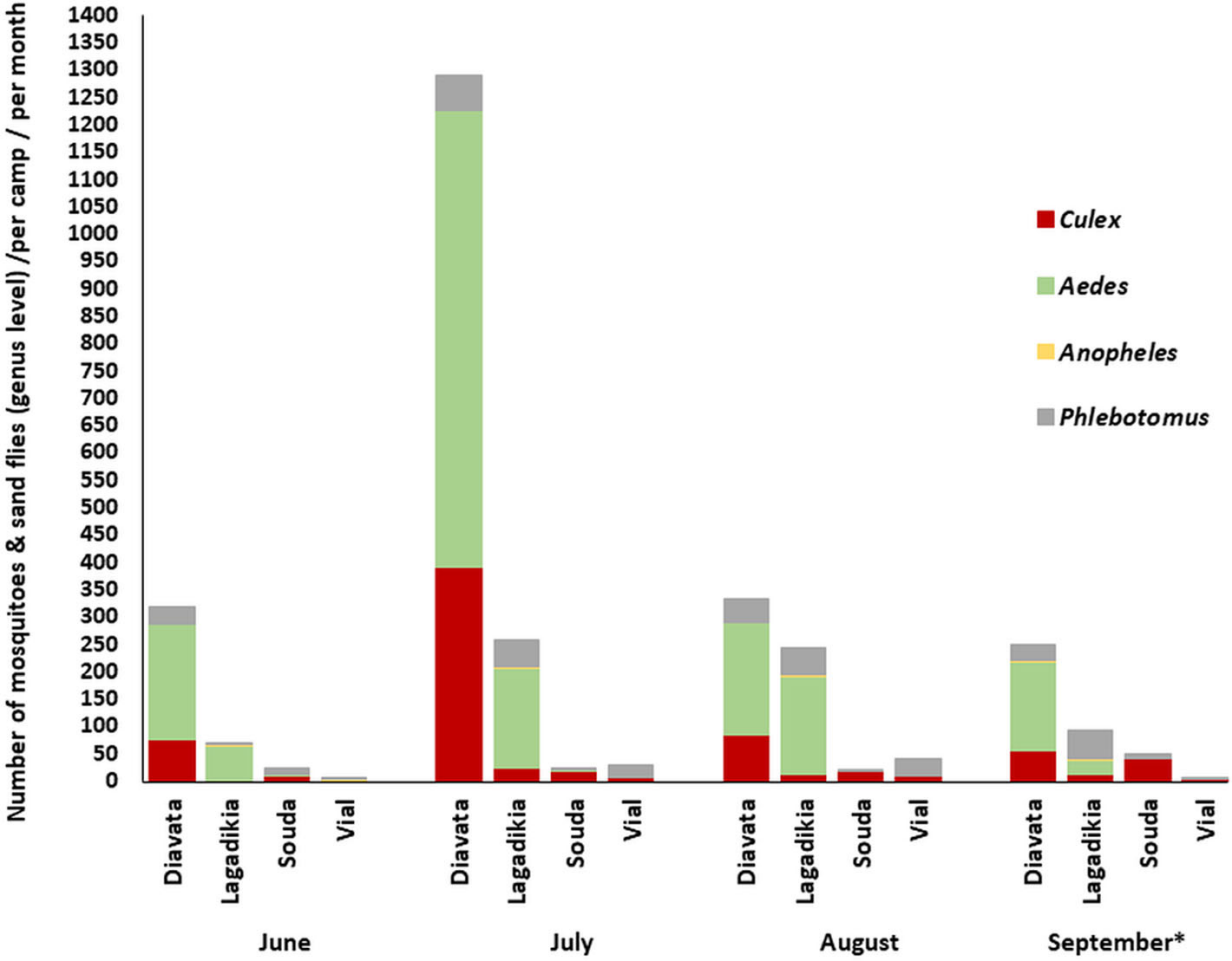
Mosquito and sand fly (genus level) population dynamics in Refugee camps, Greece. Sand fly species data displayed corresponds to male and female adult collections, mosquito species data corresponds to adult female collections. The mosquito and sand fly genera recorded are depicted with different colours. ^(^*^)^ one sample collection was conducted in September (Week 1–2) (vs) two sample collections (Week 1–2, Week 3–4) in June, July, August

In Diavata camp where *Cx. pipiens* was highly abundant the populations peaked in July (Fig. 2).

*Ae. albopictus* was detected in Diavata, Lagadikia (Thessaloniki) and Souda camp (Chios). In Lagadikia and Diavata camp *Ae. albopictus* eggs were first collected in the last week of July. Oviposition collections in Vial camp (Chios) were halted because traps were knocked down by animal activity.

The malaria vector *An. sacharovi* and the potential vector *An. maculipennis* s.s. were recorded in Lagadikia camp, albeit all at very low numbers while *An. hyrcanus* was found in Diavata camp. A single *An. claviger* specimen was collected from Vial camp (Chios).

The nuisance species *Ae. caspius* was by far the most dominant species in Diavata and Lagadikia camp (Thessaloniki) (Fig. 1, Table 1) composing 70 and 74% of the mosquito population, respectively, while *Ae. vexans* was also reported in both camps.

The sand fly species recorded were *P. perfiliewi, P. tobbi* and *P. simici* in Diavata and Lagadikia camps (Thessaloniki) and *P. neglectus, P. perfiliewi* in Souda and Vial camps (Chios). *P. perfiliewi* was the dominant species in Lagadikia, Souda and Vial camp and *P. tobbi* was the most abundant species in Diavata camp (Fig. 1, Table 1). In Lagadikia and Diavata (Thessaloniki) the sand fly populations showed very low activity in June but from then onwards population numbers increased, maintaining a relatively steady activity, with the highest population numbers in Lagadikia camp noted in early September (Fig. 2).

### Pathogen detection

All *Cx. pipiens* pools analysed were negative for *Flavivirus*/WNV presence. All *Anopheles* samples collected were negative for *P. vivax* and *P. falciparum* infections. *Leishmania donovani* and *Leishmania tropica* DNA was detected in *P. perfiliewi, P. tobbi and P. simici* specimens from Lagadikia and Diavata camps (Thessaloniki) [21].

### Vector insecticide resistance status

The pyrethroid resistance mutation L1014F, was analysed in a total of 80 *Cx. pipiens* mosquitoes and was recorded at an allele frequency ranging from 41.0 to 63.3% (Table 4). Homozygotes for the resistance mutation were detected in all camps (Additional file 1: Table S1). In a total of 46 female *Cx. pipiens* mosquitoes, no *CHS* mutations were detected (Table 4).

**Table 4:**
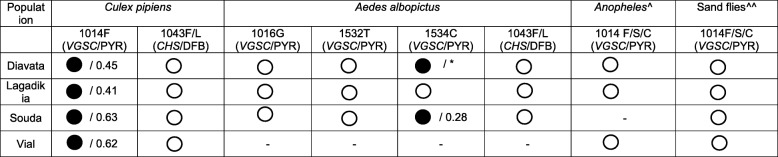
Insecticide resistance status of the main vectors sampled in the refugee camps

= Detection of Target site mutations/resistant allele frequency

= no resistance mechanisms detected

- = no samples analysed; * = Both 1534F (susceptible allele) and 1534C were found in eggs (pooled samples) from Diavata / no precise information regarding mutation frequency; *VGSC*/PYR Pyrethroid resistant mutation(s) in the Voltage gated sodium channel gene; *CHS*/DFB Diflubenzuron resistant mutations in the Chitin synthase 1 gene; ^ *Anopheles* species analysed: *An. sacharovi, An. maculipennis, An. hyrcanus*; ^^ Sand fly species analysed: *P. perfiliewi, P. tobbi, P. simici, P. neglectus*; *kdr* analysis in sand flies from Souda camp was conducted in [16]

Fourty five *Ae. albopictus* eggs and 17 *Ae. albopictus* adults were screened for the presence of pyrethroid and DFB resistance mutations at the corresponding sites 1016, 1532, 1534 (*VGSC)* and 1043 (*CHS*). All samples were homozygous for the susceptible – wild type alleles 1043I, 1016 V and 1532 T. The mutation 1534C was detected in Souda camp at an allele frequency of 28.0%, corresponding to three heterozygote specimens’ 1534F/C and a single homozygote 1534C/C. Both 1534F (susceptible allele) and 1534C were found in a pool of eggs from Diavata (Table 4).

A fragment of the *VGSC* gene was analyzed in a subset (between 14 and 24 specimens per camp) of the collected sand flies including all sand fly species detected. No pyrethroid resistance mutations were detected. Likewise no pyrethroid resistance mutations at codon 1014 were reported in the *Anopheles* specimens analyzed (Table 4).

## Discussion

Over the last five years, hundreds of thousands of refugees have sought refuge/asylum in Greece and other European countries [39]. Travelling and often living under poor conditions with limited access to healthcare, these displaced populations are vulnerable to VBDs, especially when travelling or hosted in VBD endemic regions. Entomological monitoring in four major refugee camps in Greece revealed the presence of competent disease vectors at varied levels of abundance, including vectors infected with pathogens and resistant to insecticides.

### Presence of important disease vectors in the refugee camps

Mosquito and sand fly species analyses revealed the presence of important proven and suspected disease vectors including the mosquito species; *Cx. pipiens*, *Ae. albopictus*, *An. sacharovi* and the sand flies [21] *P. tobbi*, *P. perfiliewi*, *P. simici* and *P. neglectus.* Even though there were differences in vector abundance among the four camp sites, *Cx. pipiens* s.l. mosquitoes (WNV vectors) were ubiquitous, followed by *Ae. albopictus* (dengue & Chikungunya vector) and *An. maculipennis* s.l. (malaria vector). The *Leishmania* vectors *P. perfiliewi* and *P. tobbi* were also dominant in the camp sites [21]. The observed seasonality of the recorded mosquito and sand fly vectors is in accordance with available literature from the region [11, 15] with July–September being the period with maximum activity.

### Vector population structure in relation to camp locations

High *Cx. pipiens* numbers were recorded in Diavata camp, Thessaloniki (*n* = 606), compared to the other camps, which may be attributed to the camp’s location. The camp is situated in a peri-urban area of intense industrial activity and is in close proximity to highly proliferative agricultural breeding sites (rice fields). The former possibly providing a plethora of *Cx. pipiens* breeding sites adjacent to the camp grounds and the latter resulting in the production of high *Cx. pipiens* numbers, potentially drawn towards the camp site. The highest densities of sand flies were detected in Diavata and Lagkadikia camps (Thessaloniki), with the latter being located adjacent to animal facilities (< 150 m), which are known prolific sand fly breeding sites [11]. When selecting sites for hosting refugee populations, analyses on the vector breeding potential of the candidate sites as well as their proximity in areas with a history of vector borne diseases is of high importance. Risk assessment models incorporating data on the presence/absence of confirmed and potential breeding sites, as well as historic entomological and disease surveillance data may guide the selection of appropriate sites, minimizing the potential risk for VBD transmission [40, 41]. Furthermore, the development of geo-spatial information platforms featuring real-time entomological and epidemiological data on vectors and the pathogens they transmit from the broader refugee camp regions will potentially provide well-informed risk assessments and evidence based guidance for targeted vector control.

### Presence of vector-borne pathogens

Even though all *Culex* samples tested were negative for WNV only a small number of mosquitoes were screened and, thus, may not be representative of the actual WNV pathogen circulation levels of WNV in mosquitoes within and around the survey camp sites. Consecutive follow up analyses on a larger number of specimens is essential for assessing the potential/ongoing risk for WNV transmission. To our knowledge no WNV cases have been reported to date in the study refugee camps. However, the high *Cx. pipiens* numbers and hybrid biotype representation (*Cx. pipiens molestus/pipiens* hybrids were detected at a rate of 12.5 to 33.3%) recorded in the study camp sites in conjunction with the WNV disease history in Greece [42] pose a risk for the resident camp populations and the surrounding communities. Low numbers of *Anopheles* mosquitoes were recorded in the refugee camps and no *Plasmodium* parasites were detected in the *Anopheles* samples. Nevertheless, the presence of competent malaria vectors such as *An. sacharovi* within the camp grounds indicates risk for malaria transmission upon the presence of infected hosts. The recent history of autochtonous malaria transmission in Northern Greece and the fact that many refugees/immigrants start their journeys or travel through malaria endemic regions [43] highlights the need for systematic pathogen monitoring in all camps.

High *L. donovani* and *L. tropica* infection rates were reported in *P. perfiliewi*, *P. tobbi* and *P. simici* sand flies from Lagadikia and Diavata camps (Thessaloniki), indicating high levels of parasite circulation [21]. Parasite circulation (of the two rare *Leishmania* species in Greece) appeared to be focal in the two camps (absence of parasite detection outside the camp sites) [21], indicating a minimized threat for disease transmission to the surrounding communities. The absence of *Leishmania* parasites in the analyzed sand fly samples collected nearby the Vial and Souda camps (Chios) does not prove absence of parasite circulation in these study sites. The low numbers of sand flies tested and the fact that the sampling events in Chios took place outside the camp grounds (in contrast to the camps in Thessaloniki where all samplings were conducted within) may have decreased the sampling efficiency for pathogen detection, pin pointing the need for follow up monitoring studies within the same as well as additional camp sites.

Parasite transmission from sand fly to host depends on a number of factors including the average rate of biting [44] which is primarily associated with the host exposure to sand flies seeking blood meals and is augmented in a context of overcrowding and inadequate sheltering (risk factors). These risk factors have been associated with leishmaniasis outbreaks in refugee camps/communities in Lebanon [45, 46] and are also present to an extent in the study and other refugee camps (e.g. Moria camp on Lesvos island, Greece currently hosting over 15 000 refugees while originally designed to host 3000) raising further concerns regarding disease transmission. The number of people hosted/accommodated in Lagadikia, Diavata and Vial camp doubled in 2018 compared to 2017 (Souda camp was dismantled in October 2017), with 30 and 1176 refugees/asylum seekers sleeping in tents and rub halls (inadequate vector proofing) in Lagadikia and Vial camp respectively [47].

Essential steps for the prevention and control of leishmaniasis transmission in the refugee camps include systematic active (upon consent) and passive detection of leishmaniasis in the refugee populations, effective treatment of infected patients, detection-treatment and removal of infected dogs from the camp grounds, and the replacement of tents and rubber halls with insect proof shelters. Despite the fact that the leishmaniasis burden is high in refugee populations in the Eastern Mediterranean region [43, 45], refugee population screening and clinical assessment for *Leishmania* infections is limited in European host countries [48, 49]. On the contrary, a positive step improving living conditions is an accommodation scheme providing rented housing to a small number of vulnerable asylum-seekers and refugees in Greece [50].

### Insecticide resistance status of the major disease vectors

The pyrethroid resistance mutation 1534C was detected in *Ae. albopictus* populations from Souda and Diavata camp at low frequencies, while no *kdr* mutations were detected in *Anopheles* mosquitoes and sand fly populations. On the other hand, the pyrethroid resistance mutation L1014F was detected in *Cx. pipiens* mosquitoes at an allele frequency ranging from 41.0 to 45.2% in the Thessaloniki camps (Lagadikia, Diavata) and 62.4 to 63.3% in Chios camps (Vial, Souda - out of the 27 samples analysed from Chios none were homozygous for the susceptible allele L1014) (Additional file 1: Table S1), indicating the presence of strong selection pressures. In Chios island no large scale mosquito control applications have taken place in recent years while pyrethroids are used extensively for agricultural pest control, possibly imposing strong selection pressure on the local mosquito populations, resulting in the detected mutation frequencies. This is in agreement with recent studies [15, 18, 51] where high *kdr*, *CHS* mutation frequencies and neonicotinoid resistant mosquitoes were associated with intensive agricultural insecticide applications. Further analyses investigating the presence/frequency of other polymorphisms (point mutations, locus duplications [52, 53]) at the *Cx. pipiens VGSC* codon 1014 are required in order to fully assess the 1014 locus contribution to pyrethroid resistance.

Pyrethroid based control strategies (indoor residual spraying, long lasting insecticidal nets, outdoors residual spraying) constitute the primary (or most commonly used) approach for vector management within refugee settlements around the globe [54–56]. Although the operational significance and the possible effect of the *kdr* mutation frequencies recorded in the *Cx. pipiens* and *Ae. albopictus* samples, in terms of pyrethroid suitability/efficiency for their control has to be studied, the high mutation frequencies recorded in the studied camps indicate the need for expanding the currently available methods to include novel strategies as well as novel insecticidal groups [57]. Bioassay data against the major insecticides used and genotyping of other resistance markers (e.g. levels of major detoxification genes) will further enhance our knowledge on the resistance status of the major disease vectors and facilitate the design of effective control and insecticide resistance management programs. No DFB resistance mutations were detected in *Cx. pipiens* or *Ae. albopictus* mosquitoes, indicating the suitability of the larvicide for mosquito control in the camp areas. However, as *Ae. albopictus* is a known container breeder its control via conventional larviciding applications may be extremely challenging. A plethora of container breeding sites were identified in all study camps indicating source reduction through community engagement as an important control method in the camp grounds. Educational campaigns aiming to increase the awareness of refugee populations on vector borne diseases and provide training for appropriate personal protection measures against biting arthropods needs to be integrated in the currently available healthcare support systems.

This study has potential limitations. These include: a) sample bias regarding the *Flavivirus*/WNV presence as a small number of samples was analyzed, potentially not representative of the actual WNV circulation levels in the mosquitoes, b) sand fly collection bias in the refugee camps in Chios (Vial, Souda) where the majority of collections were conducted outside the camp grounds - potentially reducing the sampling efficiency for *Leishmania* detection compared to the Thessaloniki camps where all samples were collected within the camp grounds and c) the lack of bioassay data for *Cx. pipiens* and *Ae. albopictus* against pyrethroids in order to better evaluate their resistance status and the operational impact of the detected mutation frequencies.

## Conclusions

Important disease vectors and pathogens in vectors (*Leishmania* spp.) were recorded in the refugee camps indicating a situational risk factor for disease transmission. The *Cx. pipiens* and *Ae. albopictus kdr* mutation frequencies recorded pose a potential threat against the effectiveness of pyrethroid insecticides in these settings. In contrast, pyrethroids appear suitable for the control of *Anopheles* mosquitoes and sand flies and DFB for *Cx. pipiens* and *Ae. albopictus* larvicide applications. Targeted actions ensuring adequate living conditions and the establishment of integrated vector-borne disease surveillance programs in refugee settlements are essential for protecting refugee populations against VBDs.

## Supplementary information

**Additional file 1: Table S1.** VGSC-1014 genotype and kdr mutation 1014F allele frequency in Culex pipiens populations in the study refugee camps.

## Data Availability

All data generated or analysed during this study are included in this published article (and its supplementary information files).

## Abbreviations

*AChE2*: Acetylcholinesterase-2
B.t.i.: *Bacilus thuringiensis israelensis*
CDC: Centers for Disease Control
*CHS*: Chitin Synthase
*COI*: Cytochrome oxidase subunit 1
DFB: Diflubenzuron
IRS: Indoor Residual Spraying
*ITS2*: Internal transcribed spacer two
*kdr*: Knock Down Resistance
LLINs: Long Lasting Insecticidal Nets
PCR: Polymerase chain reaction
VBD: Vector Borne Disease
*VGSC*: Voltage Gated Sodium Channel
WNV: West Nile Virus
