# Supplementary material for: Population dynamics, pathogen detection and insecticide resistance of mosquito and sand fly in refugee camps, Greece
Source: Infect Dis Poverty. 2020 Mar 18;9:30. doi: 10.1186/s40249-020-0635-4 (PMC7079361; doi:10.1186/s40249-020-0635-4)
Supplement: Supplementary file 1 — Additional file 1: Table S1. VGSC-1014 genotype and kdr mutation 1014F allele frequency in Culex pipiens populations in the study refugee camps. [file 40249_2020_635_MOESM1_ESM.docx]

Additional file 1: **Table S1**. *VGSC*-1014 genotype and *kdr* mutation 1014F allele frequency in *Culex pipiens* populations in the study refugee camps

|  |  | VGSC-1014 genotype | | |  |
| --- | --- | --- | --- | --- | --- |
| Refugee camp | *N* | Leu/Leu | Leu/Phe | Phe/Phe | Phe allele [%] |
| Diavata | 31 | 7 | 20 | 4 | 45.2 |
| Lagadikia | 22 | 5 | 16 | 1 | 41.0 |
| Souda | 15 | 0 | 11 | 4 | 63.3 |
| Vial | 12 | 0 | 9 | 3 | 62.5 |

*N* is the total number of specimens tested.

Leu/Leu = homozygotes for the wild type allele 1014L

Leu/Phe = heterozygotes; 1014L/F.

Phe/Phe = homozygotes for the mutated allele 1014F.

[%] indicates mutated allele 1014F frequency
